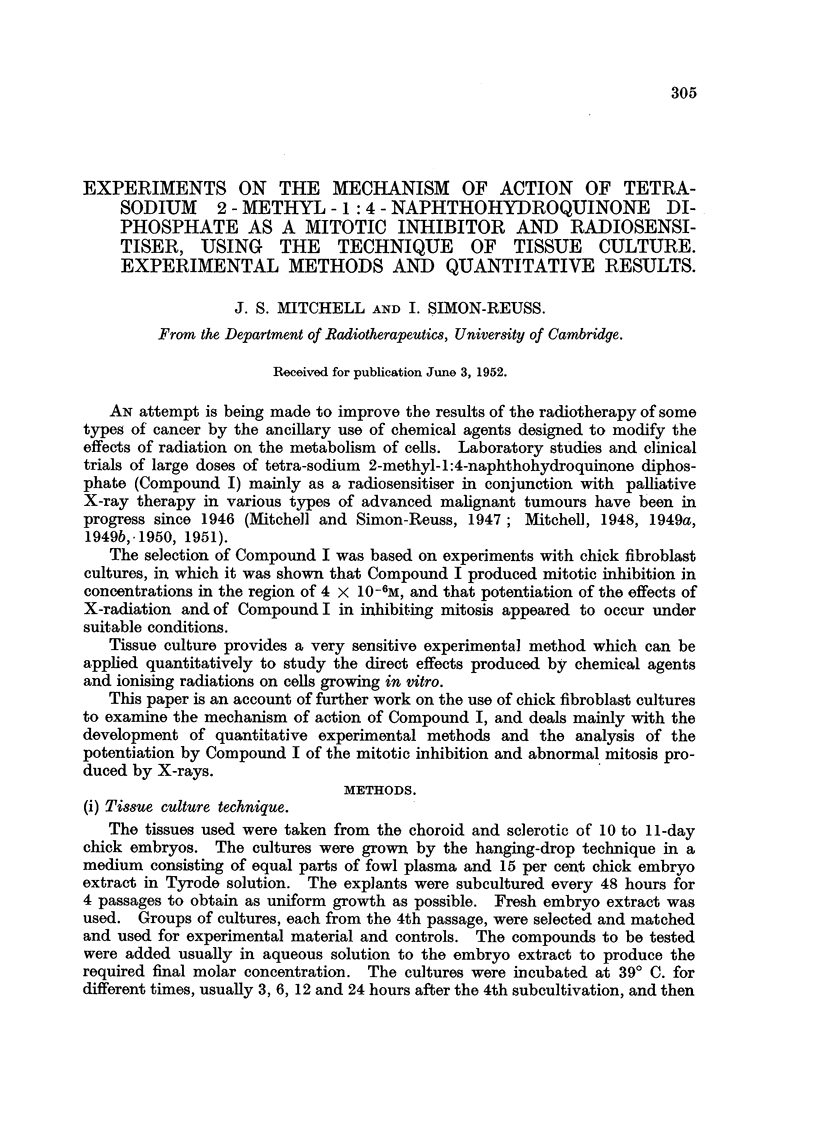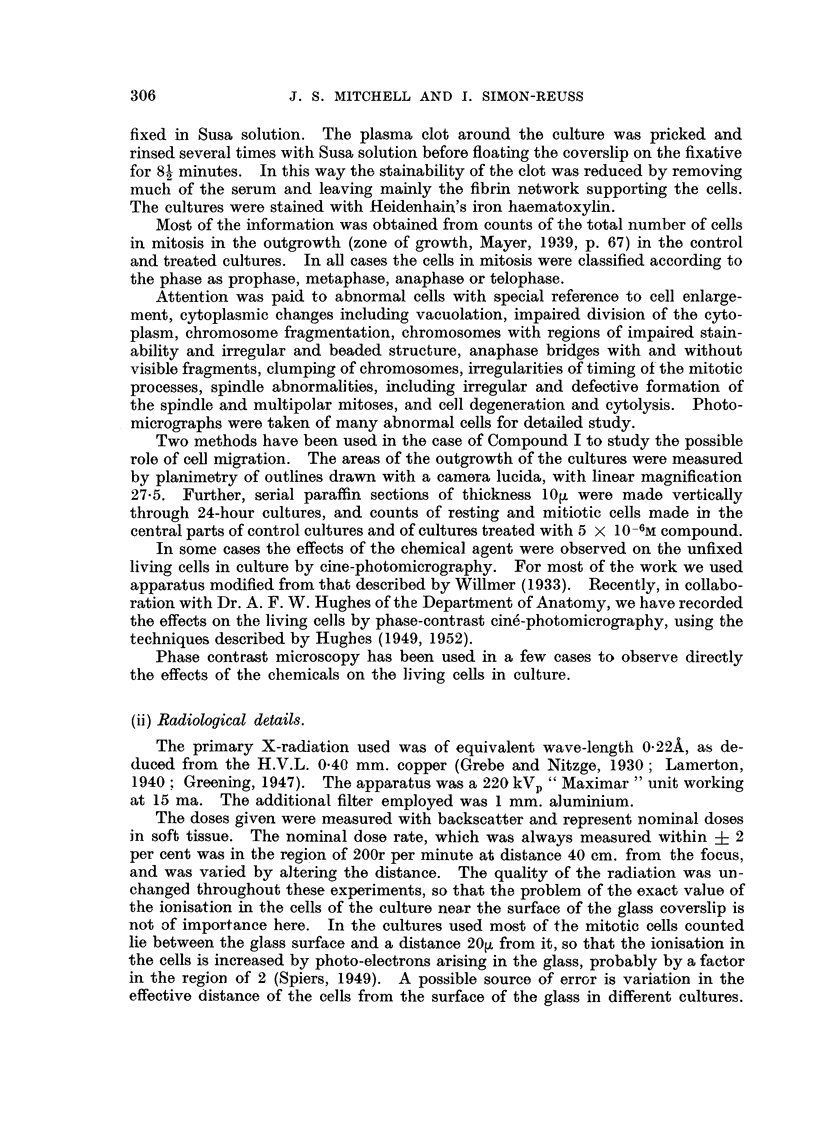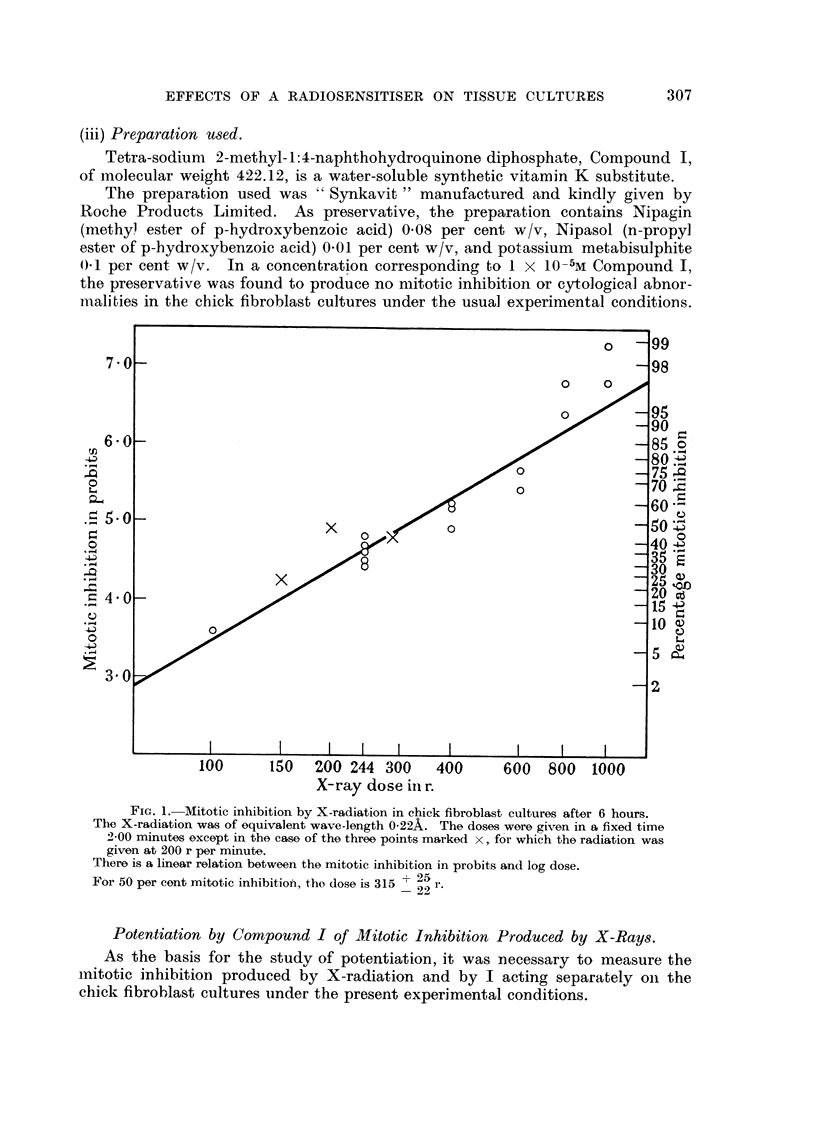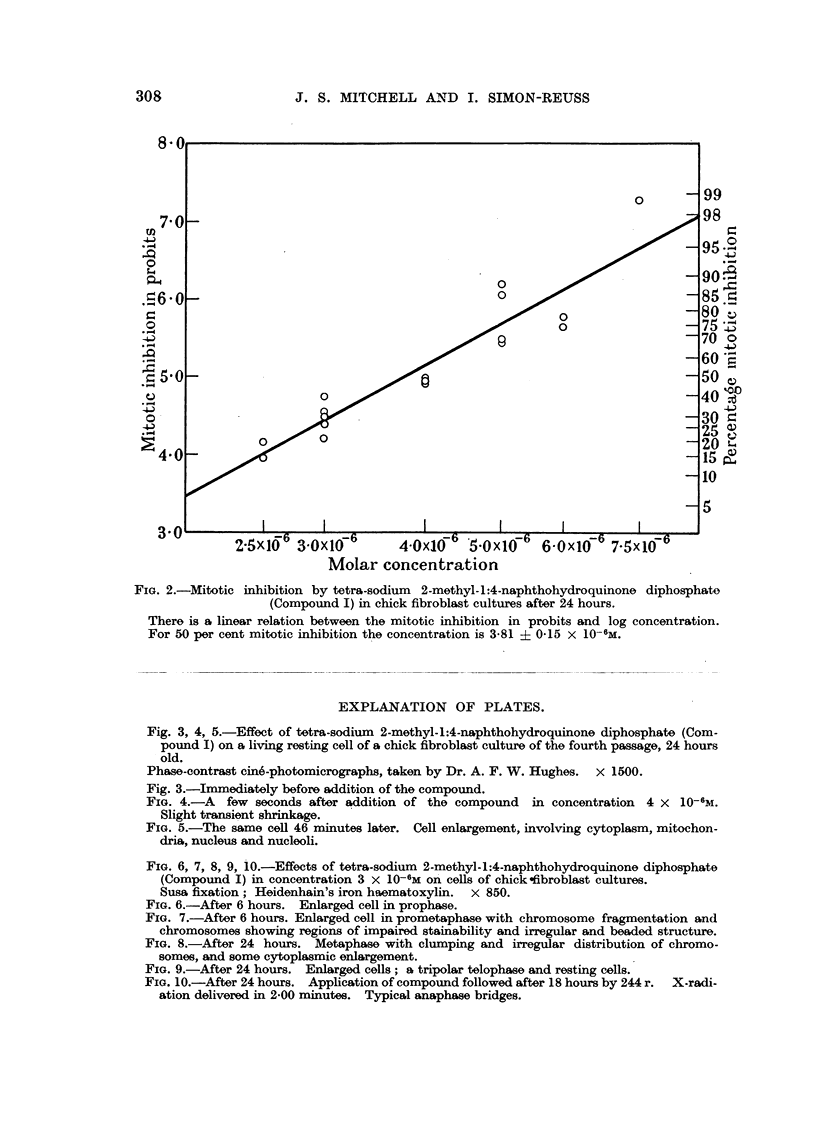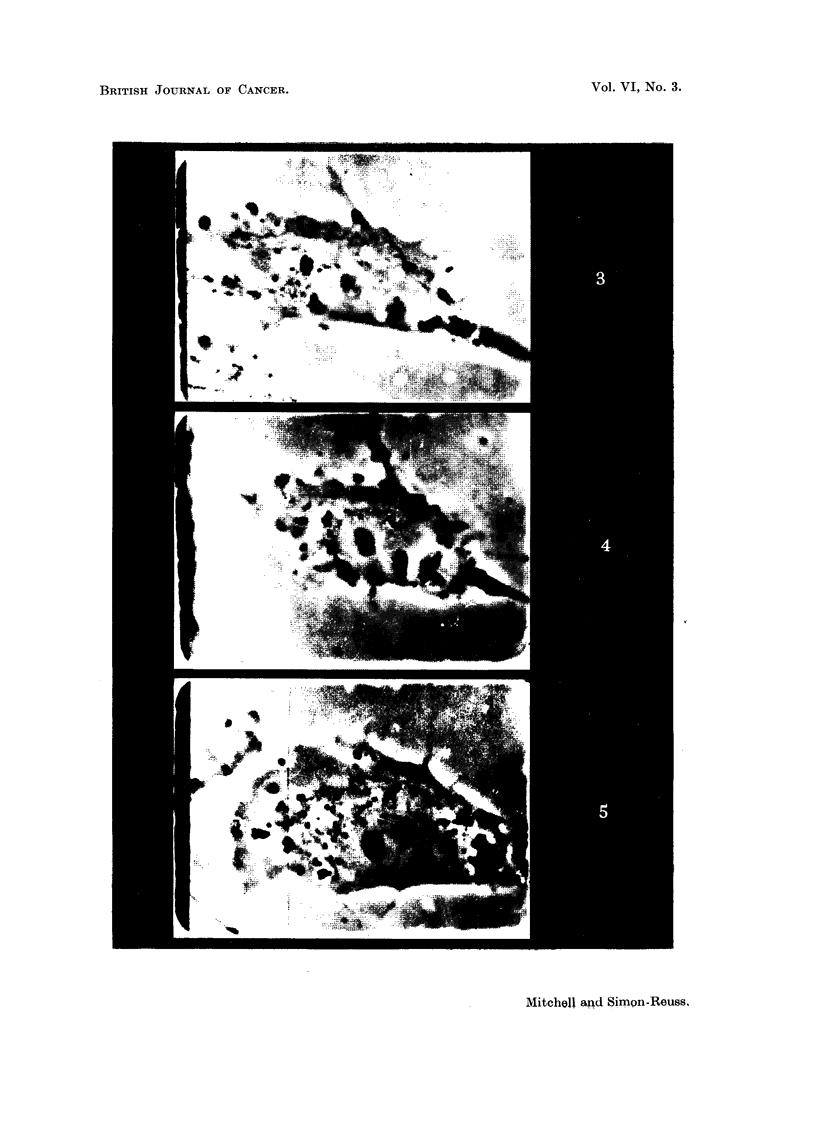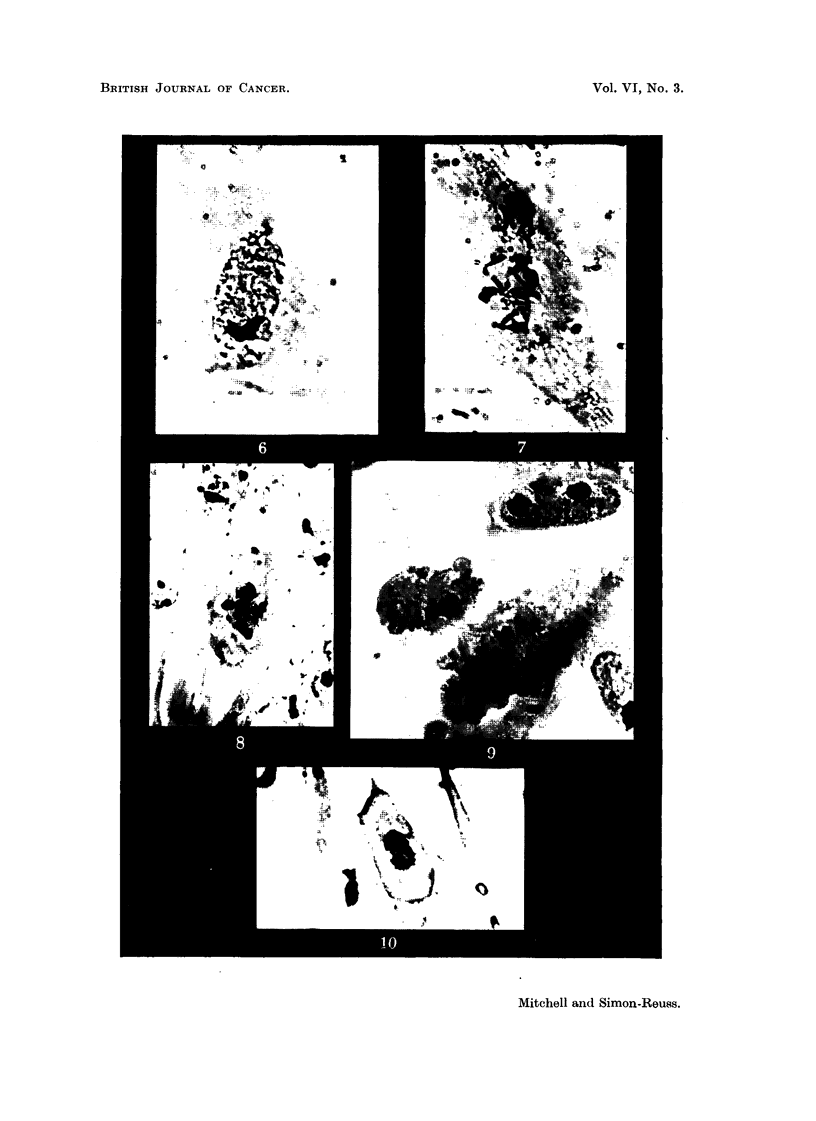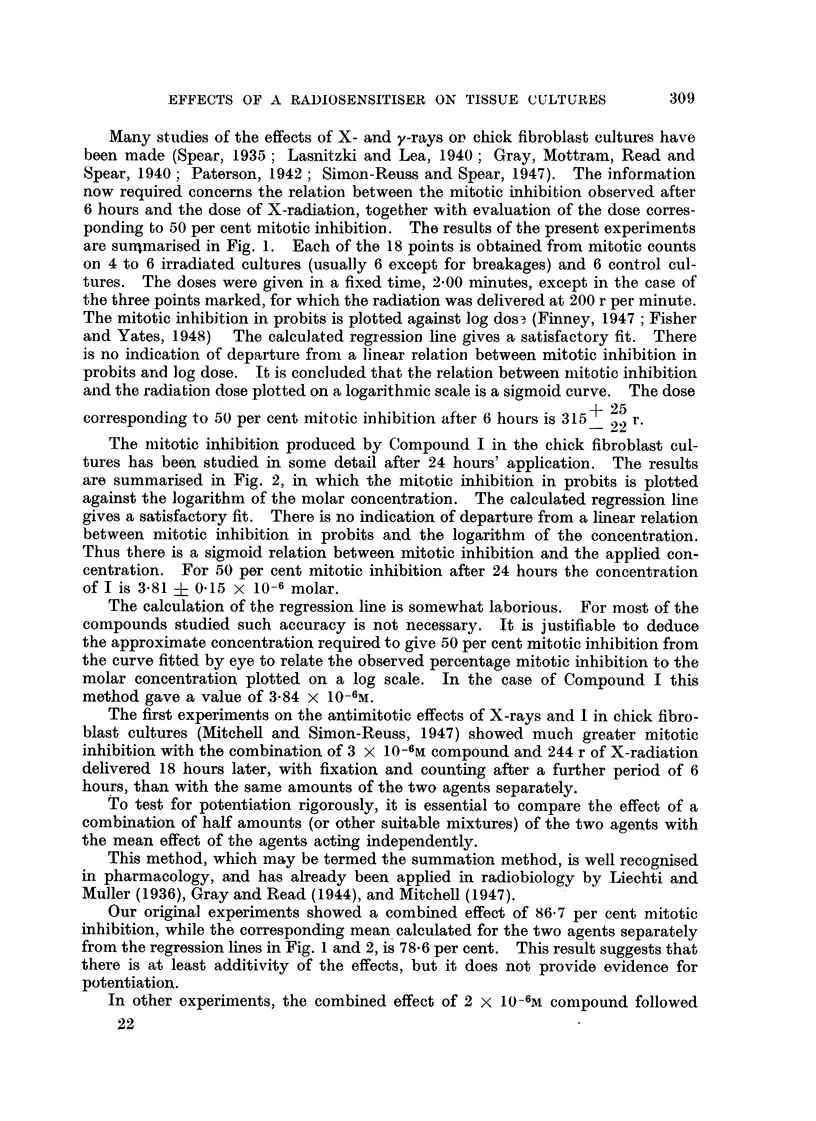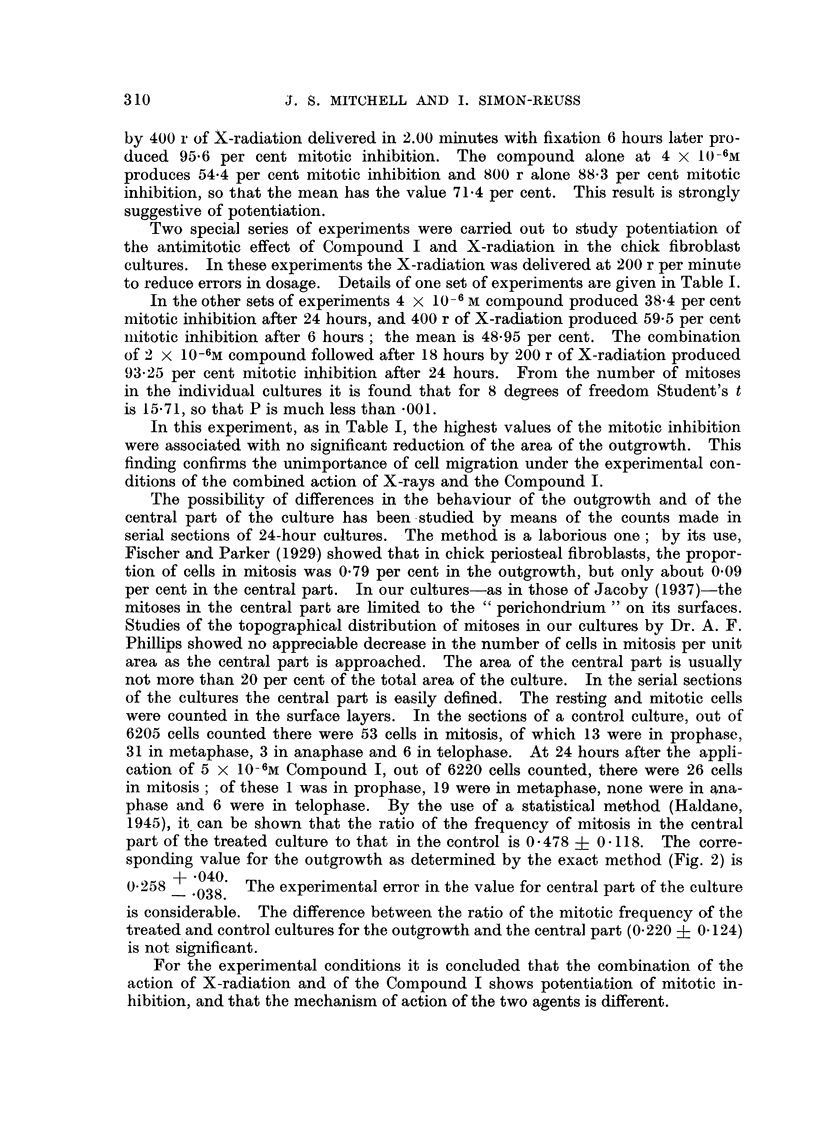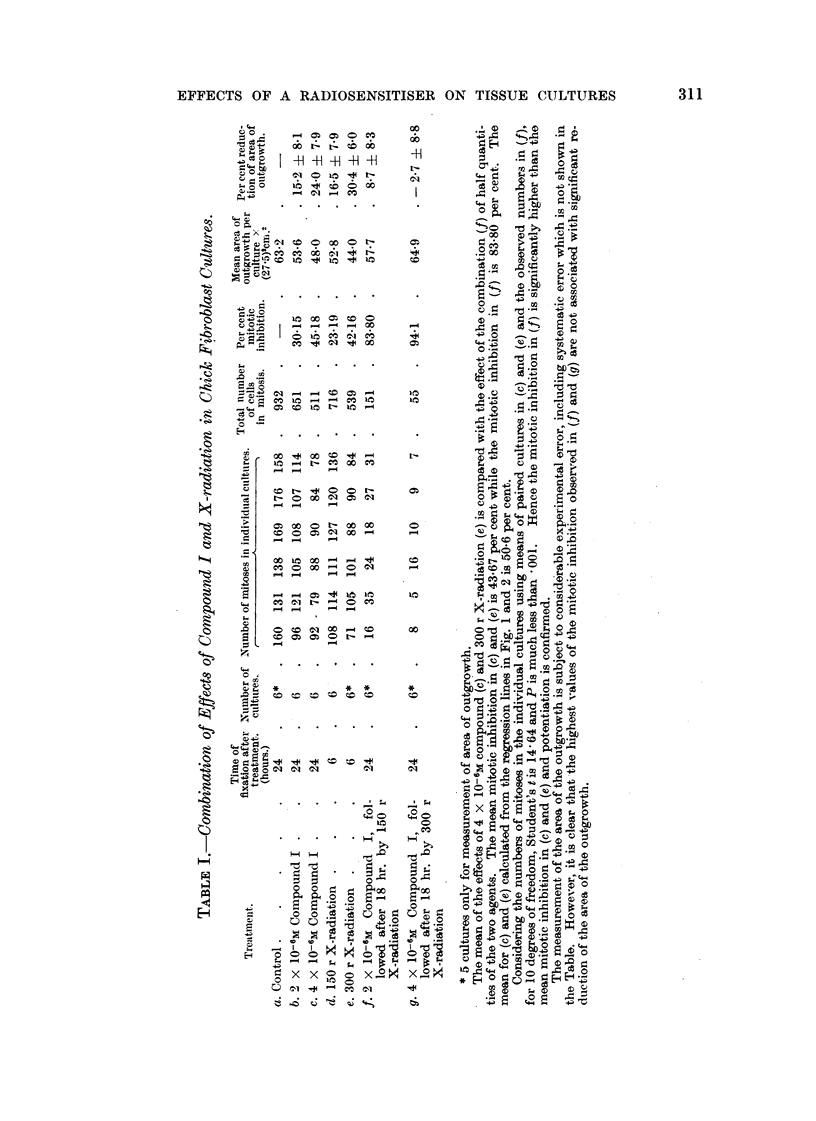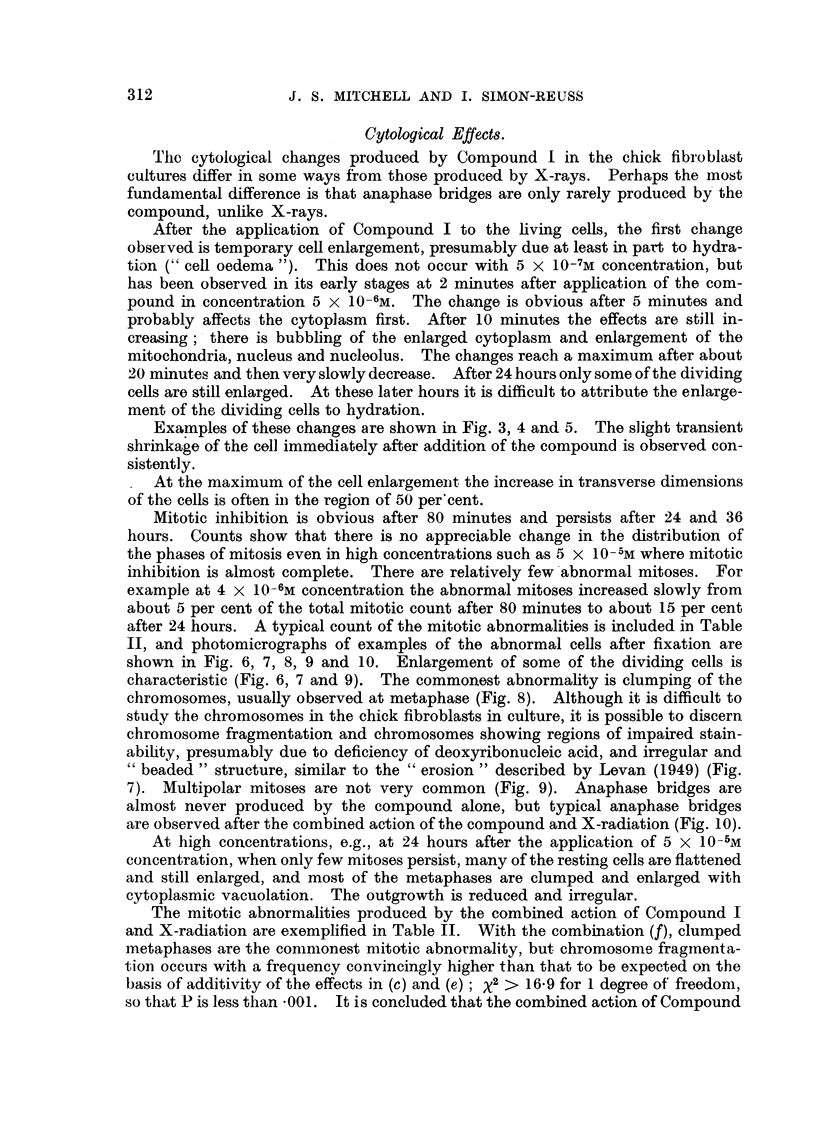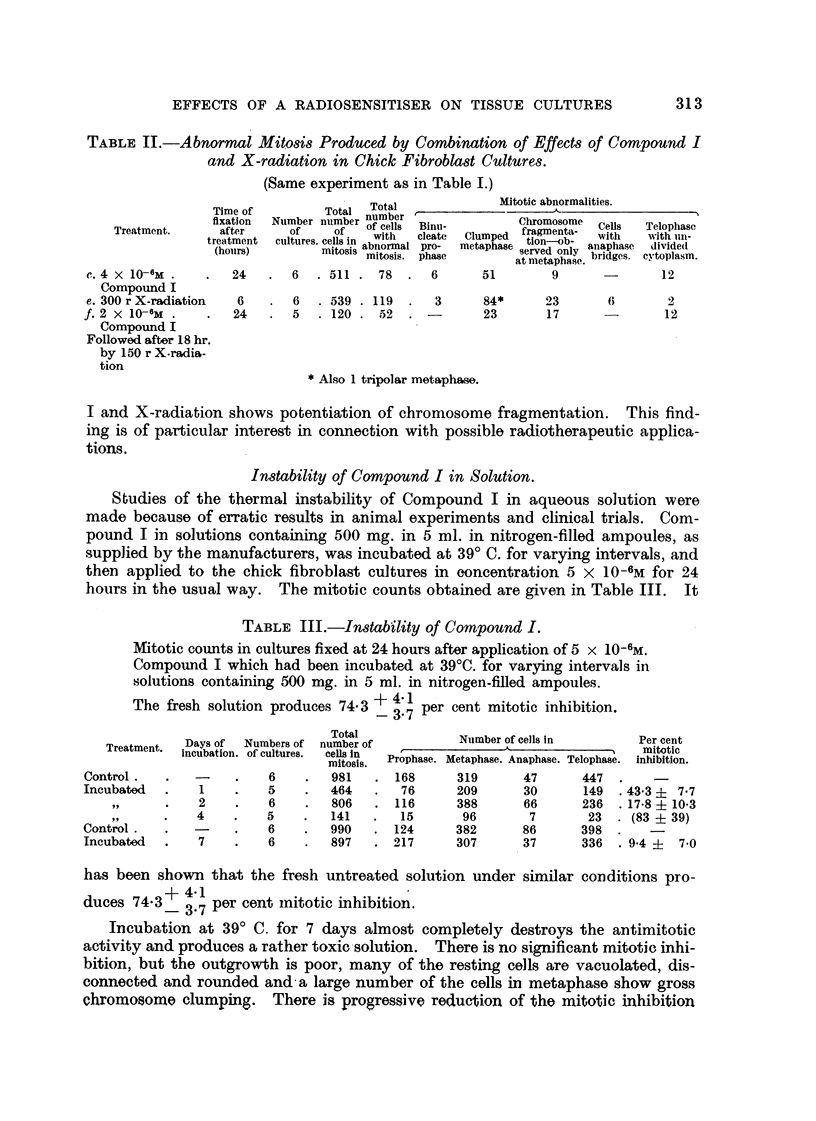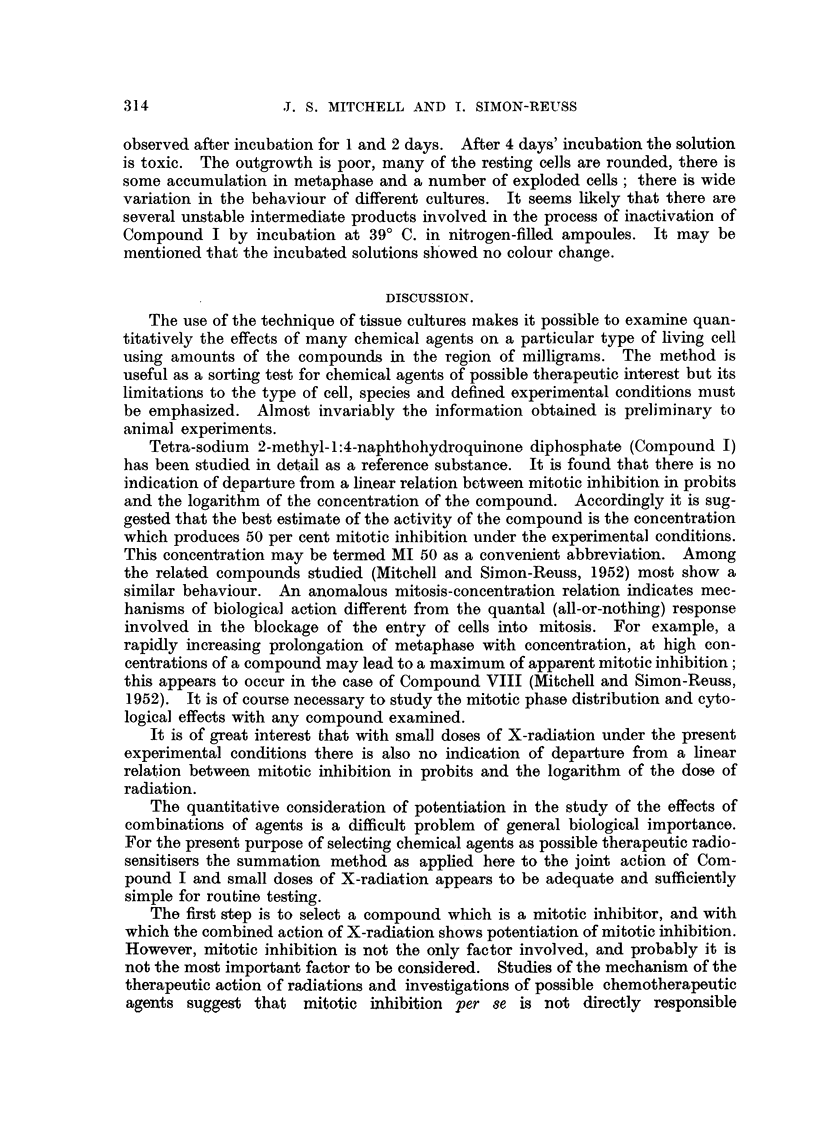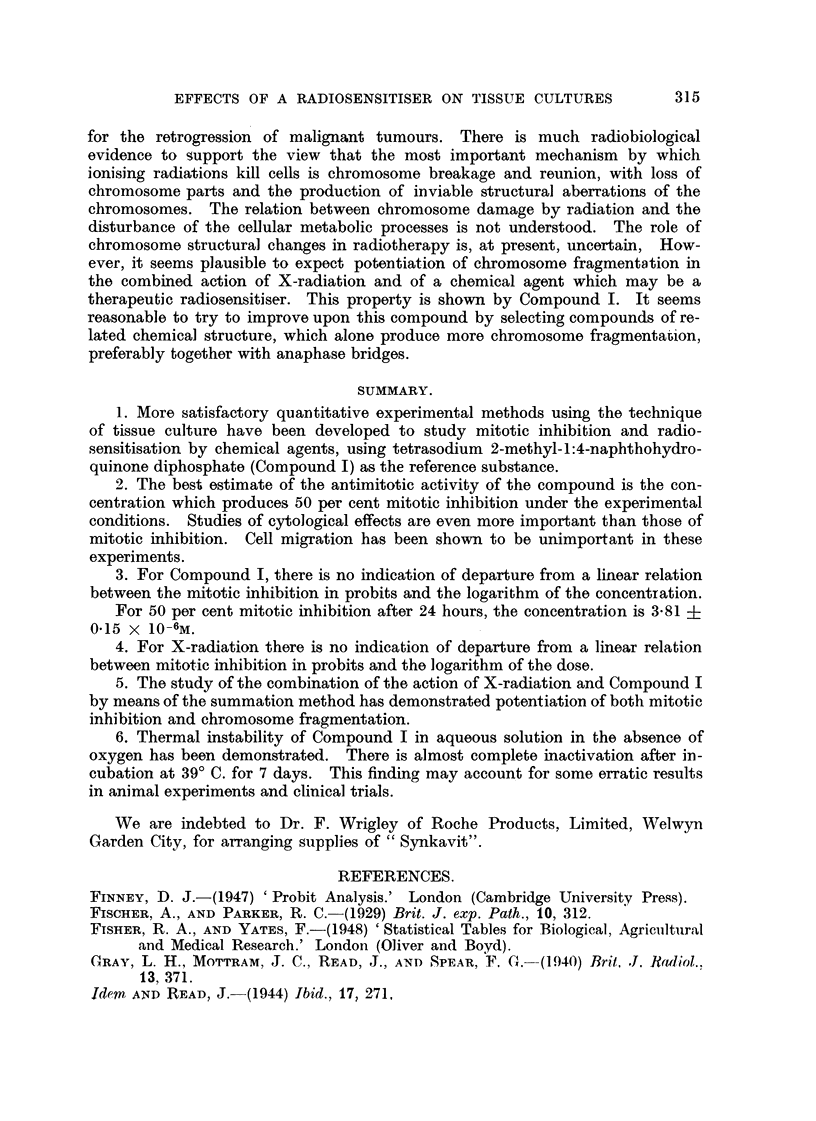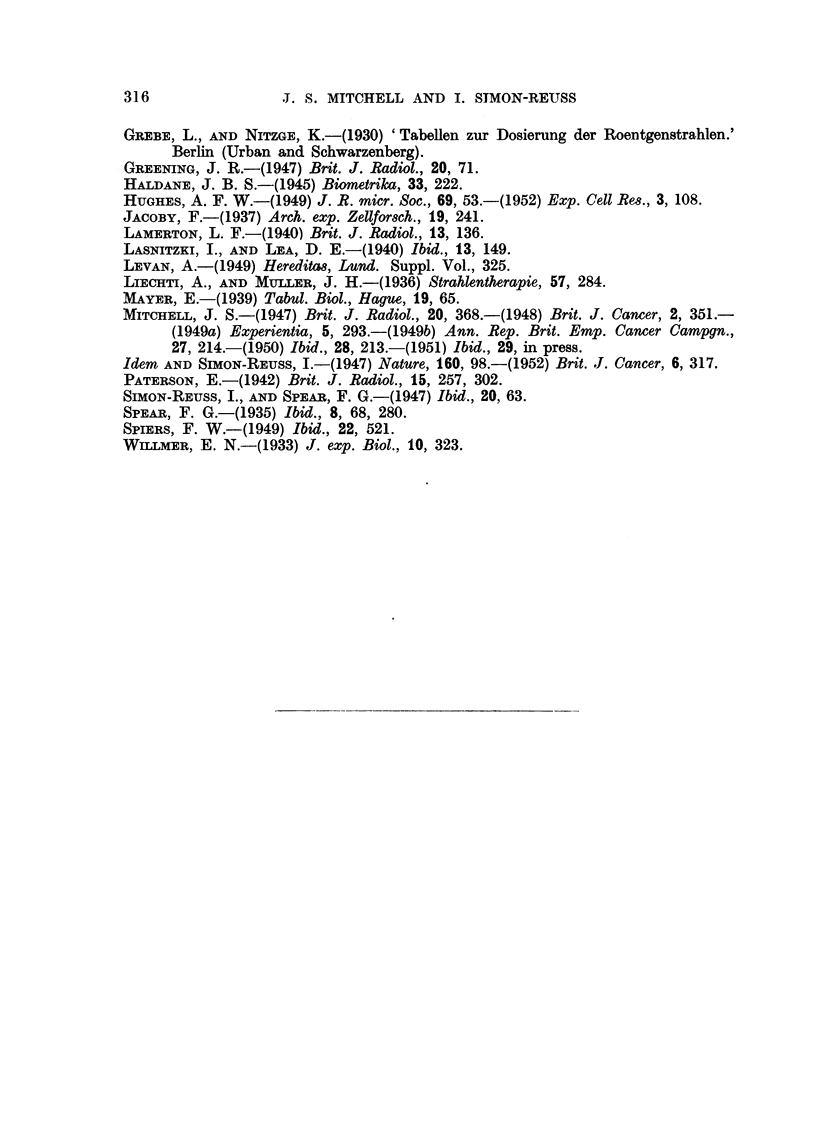# Experiments on the Mechanism of Action of Tetra-sodium 2-Methyl-1:4-Naphthohydroquinone Diphosphate as a Mitotic Inhibitor and Radiosensitiser, using the Technique of Tissue Culture. Experimental Methods and Quantitative Results

**DOI:** 10.1038/bjc.1952.35

**Published:** 1952-09

**Authors:** J. S. Mitchell, I. Simon-Reuss

## Abstract

**Images:**


### EXPERIMENTS ON THE MECHANISM OF ACTION OF TETRA-SODIUM 2-METHYL-1:4-NAPHTHOHYDROQUINONE DIPHOSPHATE AS A MITOTIC INHIBITOR AND RADIOSENSITISER, USING THE TECHNIQUE OF TISSUE CULTURE. EXPERIMENTAL METHODS AND QUANTITATIVE RESULTS.

J. S. MITCHELL AND I. SIMON-REUSS.

*From the Department of Radiotherapeutics, University of Cambridge.*

Received for publication June 3, 1952.

An attempt is being made to improve the results of the radiotherapy of some types of cancer by the ancillary use of chemical agents designed to modify the effects of radiation on the metabolism of cells. Laboratory studies and clinical trials of large doses of tetra-sodium 2-methyl-1:4-naphthohydroquinone diphosphate (Compound I) mainly as a radiosensitiser in conjunction with palliative X-ray therapy in various types of advanced malignant tumours have been in progress since 1946 (Mitchell and Simon-Reuss, 1947; Mitchell, 1948, 1949*a*, 1949*b*, 1950, 1951).

The selection of Compound I was based on experiments with chick fibroblast cultures, in which it was shown that Compound I produced mitotic inhibition in concentrations in the region of $4 \times 10^{-6}$M, and that potentiation of the effects of X-radiation and of Compound I in inhibiting mitosis appeared to occur under suitable conditions.

Tissue culture provides a very sensitive experimental method which can be applied quantitatively to study the direct effects produced by chemical agents and ionising radiations on cells growing *in vitro*.

This paper is an account of further work on the use of chick fibroblast cultures to examine the mechanism of action of Compound I, and deals mainly with the development of quantitative experimental methods and the analysis of the potentiation by Compound I of the mitotic inhibition and abnormal mitosis produced by X-rays.

### METHODS.

### (i) *Tissue culture technique.*

The tissues used were taken from the choroid and sclerotic of 10 to 11-day chick embryos. The cultures were grown by the hanging-drop technique in a medium consisting of equal parts of fowl plasma and 15 per cent chick embryo extract in Tyrode solution. The explants were subcultured every 48 hours for 4 passages to obtain as uniform growth as possible. Fresh embryo extract was used. Groups of cultures, each from the 4th passage, were selected and matched and used for experimental material and controls. The compounds to be tested were added usually in aqueous solution to the embryo extract to produce the required final molar concentration. The cultures were incubated at 39° C. for different times, usually 3, 6, 12 and 24 hours after the 4th subcultivation, and then fixed in Susa solution. The plasma clot around the culture was pricked and rinsed several times with Susa solution before floating the coverslip on the fixative for $8\frac{1}{2}$ minutes. In this way the stainability of the clot was reduced by removing much of the serum and leaving mainly the fibrin network supporting the cells. The cultures were stained with Heidenhain's iron haematoxylin.

Most of the information was obtained from counts of the total number of cells in mitosis in the outgrowth (zone of growth, Mayer, 1939, p. 67) in the control and treated cultures. In all cases the cells in mitosis were classified according to the phase as prophase, metaphase, anaphase or telophase.

Attention was paid to abnormal cells with special reference to cell enlargement, cytoplasmic changes including vacuolation, impaired division of the cytoplasm, chromosome fragmentation, chromosomes with regions of impaired stainability and irregular and beaded structure, anaphase bridges with and without visible fragments, clumping of chromosomes, irregularities of timing of the mitotic processes, spindle abnormalities, including irregular and defective formation of the spindle and multipolar mitoses, and cell degeneration and cytolysis. Photomicrographs were taken of many abnormal cells for detailed study.

Two methods have been used in the case of Compound I to study the possible role of cell migration. The areas of the outgrowth of the cultures were measured by planimetry of outlines drawn with a camera lucida, with linear magnification 27·5. Further, serial paraffin sections of thickness $10\mu$ were made vertically through 24-hour cultures, and counts of resting and mitiotic cells made in the central parts of control cultures and of cultures treated with $5 \times 10^{-6}$M compound.

In some cases the effects of the chemical agent were observed on the unfixed living cells in culture by cine-photomicrography. For most of the work we used apparatus modified from that described by Willmer (1933). Recently, in collaboration with Dr. A. F. W. Hughes of the Department of Anatomy, we have recorded the effects on the living cells by phase-contrast ciné-photomicrography, using the techniques described by Hughes (1949, 1952).

Phase contrast microscopy has been used in a few cases to observe directly the effects of the chemicals on the living cells in culture.

(ii) *Radiological details.*

The primary X-radiation used was of equivalent wave-length 0·22Å, as deduced from the H.V.L. 0·40 mm. copper (Grebe and Nitzge, 1930; Lamerton, 1940; Greening, 1947). The apparatus was a 220 $kV_p$ " Maximar " unit working at 15 ma. The additional filter employed was 1 mm. aluminium.

The doses given were measured with backscatter and represent nominal doses in soft tissue. The nominal dose rate, which was always measured within $\pm$ 2 per cent was in the region of 200r per minute at distance 40 cm. from the focus, and was varied by altering the distance. The quality of the radiation was unchanged throughout these experiments, so that the problem of the exact value of the ionisation in the cells of the culture near the surface of the glass coverslip is not of importance here. In the cultures used most of the mitotic cells counted lie between the glass surface and a distance $20\mu$ from it, so that the ionisation in the cells is increased by photo-electrons arising in the glass, probably by a factor in the region of 2 (Spiers, 1949). A possible source of error is variation in the effective distance of the cells from the surface of the glass in different cultures.

(iii) *Preparation used.*

Tetra-sodium 2-methyl-1:4-naphthohydroquinone diphosphate, Compound I, of molecular weight 422.12, is a water-soluble synthetic vitamin K substitute.

The preparation used was " Synkavit " manufactured and kindly given by Roche Products Limited. As preservative, the preparation contains Nipagin (methyl ester of p-hydroxybenzoic acid) 0·08 per cent w/v, Nipasol (n-propyl ester of p-hydroxybenzoic acid) 0·01 per cent w/v, and potassium metabisulphite 0·1 per cent w/v. In a concentration corresponding to $1 \times 10^{-5}$M Compound I, the preservative was found to produce no mitotic inhibition or cytological abnormalities in the chick fibroblast cultures under the usual experimental conditions.

FIG. 1.—Mitotic inhibition by X-radiation in chick fibroblast cultures after 6 hours. The X-radiation was of equivalent wave-length 0·22Å. The doses were given in a fixed time 2·00 minutes except in the case of the three points marked ×, for which the radiation was given at 200 r per minute.

There is a linear relation between the mitotic inhibition in probits and log dose.

For 50 per cent mitotic inhibition, the dose is $315 \begin{smallmatrix} + 25 \\ - 22 \end{smallmatrix}$ r.

*Potentiation by Compound I of Mitotic Inhibition Produced by X-Rays.*

As the basis for the study of potentiation, it was necessary to measure the mitotic inhibition produced by X-radiation and by I acting separately on the chick fibroblast cultures under the present experimental conditions.

FIG. 2.—Mitotic inhibition by tetra-sodium 2-methyl-1:4-naphthohydroquinone diphosphate (Compound I) in chick fibroblast cultures after 24 hours.

There is a linear relation between the mitotic inhibition in probits and log concentration. For 50 per cent mitotic inhibition the concentration is $3{\cdot}81 \pm 0{\cdot}15 \times 10^{-6}$M.

### EXPLANATION OF PLATES.

Fig. 3, 4, 5.—Effect of tetra-sodium 2-methyl-1:4-naphthohydroquinone diphosphate (Compound I) on a living resting cell of a chick fibroblast culture of the fourth passage, 24 hours old.

Phase-contrast ciné-photomicrographs, taken by Dr. A. F. W. Hughes. × 1500.

Fig. 3.—Immediately before addition of the compound.

FIG. 4.—A few seconds after addition of the compound in concentration $4 \times 10^{-6}$M. Slight transient shrinkage.

FIG. 5.—The same cell 46 minutes later. Cell enlargement, involving cytoplasm, mitochondria, nucleus and nucleoli.

FIG. 6, 7, 8, 9, 10.—Effects of tetra-sodium 2-methyl-1:4-naphthohydroquinone diphosphate (Compound I) in concentration $3 \times 10^{-6}$M on cells of chick fibroblast cultures.

Susa fixation ; Heidenhain's iron haematoxylin. × 850.

FIG. 6.—After 6 hours. Enlarged cell in prophase.

FIG. 7.—After 6 hours. Enlarged cell in prometaphase with chromosome fragmentation and chromosomes showing regions of impaired stainability and irregular and beaded structure.

FIG. 8.—After 24 hours. Metaphase with clumping and irregular distribution of chromosomes, and some cytoplasmic enlargement.

FIG. 9.—After 24 hours. Enlarged cells ; a tripolar telophase and resting cells.

FIG. 10.—After 24 hours. Application of compound followed after 18 hours by 244 r. X-radiation delivered in 2·00 minutes. Typical anaphase bridges.

Mitchell and Simon-Reuss.

Many studies of the effects of X- and $\gamma$-rays on chick fibroblast cultures have been made (Spear, 1935 ; Lasnitzki and Lea, 1940 ; Gray, Mottram, Read and Spear, 1940 ; Paterson, 1942 ; Simon-Reuss and Spear, 1947). The information now required concerns the relation between the mitotic inhibition observed after 6 hours and the dose of X-radiation, together with evaluation of the dose corresponding to 50 per cent mitotic inhibition. The results of the present experiments are summarised in Fig. 1. Each of the 18 points is obtained from mitotic counts on 4 to 6 irradiated cultures (usually 6 except for breakages) and 6 control cultures. The doses were given in a fixed time, 2·00 minutes, except in the case of the three points marked, for which the radiation was delivered at 200 r per minute. The mitotic inhibition in probits is plotted against log dose (Finney, 1947 ; Fisher and Yates, 1948) The calculated regression line gives a satisfactory fit. There is no indication of departure from a linear relation between mitotic inhibition in probits and log dose. It is concluded that the relation between mitotic inhibition and the radiation dose plotted on a logarithmic scale is a sigmoid curve. The dose corresponding to 50 per cent mitotic inhibition after 6 hours is $315^{+25}_{-22}$ r.

The mitotic inhibition produced by Compound I in the chick fibroblast cultures has been studied in some detail after 24 hours' application. The results are summarised in Fig. 2, in which the mitotic inhibition in probits is plotted against the logarithm of the molar concentration. The calculated regression line gives a satisfactory fit. There is no indication of departure from a linear relation between mitotic inhibition in probits and the logarithm of the concentration. Thus there is a sigmoid relation between mitotic inhibition and the applied concentration. For 50 per cent mitotic inhibition after 24 hours the concentration of I is $3{\cdot}81 \pm 0{\cdot}15 \times 10^{-6}$ molar.

The calculation of the regression line is somewhat laborious. For most of the compounds studied such accuracy is not necessary. It is justifiable to deduce the approximate concentration required to give 50 per cent mitotic inhibition from the curve fitted by eye to relate the observed percentage mitotic inhibition to the molar concentration plotted on a log scale. In the case of Compound I this method gave a value of $3{\cdot}84 \times 10^{-6}$M.

The first experiments on the antimitotic effects of X-rays and I in chick fibroblast cultures (Mitchell and Simon-Reuss, 1947) showed much greater mitotic inhibition with the combination of $3 \times 10^{-6}$M compound and 244 r of X-radiation delivered 18 hours later, with fixation and counting after a further period of 6 hours, than with the same amounts of the two agents separately.

To test for potentiation rigorously, it is essential to compare the effect of a combination of half amounts (or other suitable mixtures) of the two agents with the mean effect of the agents acting independently.

This method, which may be termed the summation method, is well recognised in pharmacology, and has already been applied in radiobiology by Liechti and Muller (1936), Gray and Read (1944), and Mitchell (1947).

Our original experiments showed a combined effect of 86·7 per cent mitotic inhibition, while the corresponding mean calculated for the two agents separately from the regression lines in Fig. 1 and 2, is 78·6 per cent. This result suggests that there is at least additivity of the effects, but it does not provide evidence for potentiation.

In other experiments, the combined effect of $2 \times 10^{-6}$M compound followed by 400 r of X-radiation delivered in 2.00 minutes with fixation 6 hours later produced 95·6 per cent mitotic inhibition. The compound alone at $4 \times 10^{-6}$M produces 54·4 per cent mitotic inhibition and 800 r alone 88·3 per cent mitotic inhibition, so that the mean has the value 71·4 per cent. This result is strongly suggestive of potentiation.

Two special series of experiments were carried out to study potentiation of the antimitotic effect of Compound I and X-radiation in the chick fibroblast cultures. In these experiments the X-radiation was delivered at 200 r per minute to reduce errors in dosage. Details of one set of experiments are given in Table I.

In the other sets of experiments $4 \times 10^{-6}$ M compound produced 38·4 per cent mitotic inhibition after 24 hours, and 400 r of X-radiation produced 59·5 per cent mitotic inhibition after 6 hours ; the mean is 48·95 per cent. The combination of $2 \times 10^{-6}$M compound followed after 18 hours by 200 r of X-radiation produced 93·25 per cent mitotic inhibition after 24 hours. From the number of mitoses in the individual cultures it is found that for 8 degrees of freedom Student's $t$ is 15·71, so that P is much less than ·001.

In this experiment, as in Table I, the highest values of the mitotic inhibition were associated with no significant reduction of the area of the outgrowth. This finding confirms the unimportance of cell migration under the experimental conditions of the combined action of X-rays and the Compound I.

The possibility of differences in the behaviour of the outgrowth and of the central part of the culture has been studied by means of the counts made in serial sections of 24-hour cultures. The method is a laborious one ; by its use, Fischer and Parker (1929) showed that in chick periosteal fibroblasts, the proportion of cells in mitosis was 0·79 per cent in the outgrowth, but only about 0·09 per cent in the central part. In our cultures—as in those of Jacoby (1937)—the mitoses in the central part are limited to the " perichondrium " on its surfaces. Studies of the topographical distribution of mitoses in our cultures by Dr. A. F. Phillips showed no appreciable decrease in the number of cells in mitosis per unit area as the central part is approached. The area of the central part is usually not more than 20 per cent of the total area of the culture. In the serial sections of the cultures the central part is easily defined. The resting and mitotic cells were counted in the surface layers. In the sections of a control culture, out of 6205 cells counted there were 53 cells in mitosis, of which 13 were in prophase, 31 in metaphase, 3 in anaphase and 6 in telophase. At 24 hours after the application of $5 \times 10^{-6}$M Compound I, out of 6220 cells counted, there were 26 cells in mitosis ; of these 1 was in prophase, 19 were in metaphase, none were in anaphase and 6 were in telophase. By the use of a statistical method (Haldane, 1945), it can be shown that the ratio of the frequency of mitosis in the central part of the treated culture to that in the control is $0·478 \pm 0·118$. The corresponding value for the outgrowth as determined by the exact method (Fig. 2) is $0·258 {+ ·040. \atop - ·038.}$ The experimental error in the value for central part of the culture is considerable. The difference between the ratio of the mitotic frequency of the treated and control cultures for the outgrowth and the central part ($0·220 \pm 0·124$) is not significant.

For the experimental conditions it is concluded that the combination of the action of X-radiation and of the Compound I shows potentiation of mitotic inhibition, and that the mechanism of action of the two agents is different.

### *Cytological Effects.*

The cytological changes produced by Compound I in the chick fibroblast cultures differ in some ways from those produced by X-rays. Perhaps the most fundamental difference is that anaphase bridges are only rarely produced by the compound, unlike X-rays.

After the application of Compound I to the living cells, the first change observed is temporary cell enlargement, presumably due at least in part to hydration ("cell oedema"). This does not occur with $5 \times 10^{-7}$M concentration, but has been observed in its early stages at 2 minutes after application of the compound in concentration $5 \times 10^{-6}$M. The change is obvious after 5 minutes and probably affects the cytoplasm first. After 10 minutes the effects are still increasing; there is bubbling of the enlarged cytoplasm and enlargement of the mitochondria, nucleus and nucleolus. The changes reach a maximum after about 20 minutes and then very slowly decrease. After 24 hours only some of the dividing cells are still enlarged. At these later hours it is difficult to attribute the enlargement of the dividing cells to hydration.

Examples of these changes are shown in Fig. 3, 4 and 5. The slight transient shrinkage of the cell immediately after addition of the compound is observed consistently.

At the maximum of the cell enlargement the increase in transverse dimensions of the cells is often in the region of 50 per cent.

Mitotic inhibition is obvious after 80 minutes and persists after 24 and 36 hours. Counts show that there is no appreciable change in the distribution of the phases of mitosis even in high concentrations such as $5 \times 10^{-5}$M where mitotic inhibition is almost complete. There are relatively few abnormal mitoses. For example at $4 \times 10^{-6}$M concentration the abnormal mitoses increased slowly from about 5 per cent of the total mitotic count after 80 minutes to about 15 per cent after 24 hours. A typical count of the mitotic abnormalities is included in Table II, and photomicrographs of examples of the abnormal cells after fixation are shown in Fig. 6, 7, 8, 9 and 10. Enlargement of some of the dividing cells is characteristic (Fig. 6, 7 and 9). The commonest abnormality is clumping of the chromosomes, usually observed at metaphase (Fig. 8). Although it is difficult to study the chromosomes in the chick fibroblasts in culture, it is possible to discern chromosome fragmentation and chromosomes showing regions of impaired stainability, presumably due to deficiency of deoxyribonucleic acid, and irregular and "beaded" structure, similar to the "erosion" described by Levan (1949) (Fig. 7). Multipolar mitoses are not very common (Fig. 9). Anaphase bridges are almost never produced by the compound alone, but typical anaphase bridges are observed after the combined action of the compound and X-radiation (Fig. 10).

At high concentrations, e.g., at 24 hours after the application of $5 \times 10^{-5}$M concentration, when only few mitoses persist, many of the resting cells are flattened and still enlarged, and most of the metaphases are clumped and enlarged with cytoplasmic vacuolation. The outgrowth is reduced and irregular.

The mitotic abnormalities produced by the combined action of Compound I and X-radiation are exemplified in Table II. With the combination (*f*), clumped metaphases are the commonest mitotic abnormality, but chromosome fragmentation occurs with a frequency convincingly higher than that to be expected on the basis of additivity of the effects in (*c*) and (*e*); $\chi^2 > 16{\cdot}9$ for 1 degree of freedom, so that P is less than ·001. It is concluded that the combined action of Compound

TABLE II.—*Abnormal Mitosis Produced by Combination of Effects of Compound I and X-radiation in Chick Fibroblast Cultures.*

(Same experiment as in Table I.)

| Treatment. | Time of fixation after treatment (hours) | Number of cultures. | Total number of cells in mitosis | Total number of cells with abnormal mitosis. | Mitotic abnormalities. Binucleate prophase | Clumped metaphase | Chromosome fragmentation—observed only at metaphase. | Cells with anaphase bridges. | Telophase with undivided cytoplasm. |
|---|---|---|---|---|---|---|---|---|---|
| c. 4 × $10^{-6}$M . Compound I | 24 | 6 | 511 | 78 | 6 | 51 | 9 | — | 12 |
| e. 300 r X-radiation | 6 | 6 | 539 | 119 | 3 | 84* | 23 | 6 | 2 |
| f. 2 × $10^{-6}$M . Compound I Followed after 18 hr. by 150 r X-radiation | 24 | 5 | 120 | 52 | — | 23 | 17 | — | 12 |

* Also 1 tripolar metaphase.

I and X-radiation shows potentiation of chromosome fragmentation. This finding is of particular interest in connection with possible radiotherapeutic applications.

### *Instability of Compound I in Solution.*

Studies of the thermal instability of Compound I in aqueous solution were made because of erratic results in animal experiments and clinical trials. Compound I in solutions containing 500 mg. in 5 ml. in nitrogen-filled ampoules, as supplied by the manufacturers, was incubated at 39° C. for varying intervals, and then applied to the chick fibroblast cultures in concentration 5 × $10^{-6}$M for 24 hours in the usual way. The mitotic counts obtained are given in Table III. It

TABLE III.—*Instability of Compound I.*

Mitotic counts in cultures fixed at 24 hours after application of 5 × $10^{-6}$M. Compound I which had been incubated at 39°C. for varying intervals in solutions containing 500 mg. in 5 ml. in nitrogen-filled ampoules.

The fresh solution produces $74{\cdot}3\,{}^{+\,4\cdot1}_{-\,3\cdot7}$ per cent mitotic inhibition.

| Treatment. | Days of incubation. | Numbers of cultures. | Total number of cells in mitosis. | Number of cells in Prophase. | Metaphase. | Anaphase. | Telophase. | Per cent mitotic inhibition. |
|---|---|---|---|---|---|---|---|---|
| Control . | — | 6 | 981 | 168 | 319 | 47 | 447 | — |
| Incubated | 1 | 5 | 464 | 76 | 209 | 30 | 149 | 43·3 ± 7·7 |
| ,, | 2 | 6 | 806 | 116 | 388 | 66 | 236 | 17·8 ± 10·3 |
| ,, | 4 | 5 | 141 | 15 | 96 | 7 | 23 | (83 ± 39) |
| Control . | — | 6 | 990 | 124 | 382 | 86 | 398 | — |
| Incubated | 7 | 6 | 897 | 217 | 307 | 37 | 336 | 9·4 ± 7·0 |

has been shown that the fresh untreated solution under similar conditions produces $74{\cdot}3\,{}^{+\,4\cdot1}_{-\,3\cdot7}$ per cent mitotic inhibition.

Incubation at 39° C. for 7 days almost completely destroys the antimitotic activity and produces a rather toxic solution. There is no significant mitotic inhibition, but the outgrowth is poor, many of the resting cells are vacuolated, disconnected and rounded and a large number of the cells in metaphase show gross chromosome clumping. There is progressive reduction of the mitotic inhibition observed after incubation for 1 and 2 days. After 4 days' incubation the solution is toxic. The outgrowth is poor, many of the resting cells are rounded, there is some accumulation in metaphase and a number of exploded cells ; there is wide variation in the behaviour of different cultures. It seems likely that there are several unstable intermediate products involved in the process of inactivation of Compound I by incubation at 39° C. in nitrogen-filled ampoules. It may be mentioned that the incubated solutions showed no colour change.

### DISCUSSION.

The use of the technique of tissue cultures makes it possible to examine quantitatively the effects of many chemical agents on a particular type of living cell using amounts of the compounds in the region of milligrams. The method is useful as a sorting test for chemical agents of possible therapeutic interest but its limitations to the type of cell, species and defined experimental conditions must be emphasized. Almost invariably the information obtained is preliminary to animal experiments.

Tetra-sodium 2-methyl-1:4-naphthohydroquinone diphosphate (Compound I) has been studied in detail as a reference substance. It is found that there is no indication of departure from a linear relation between mitotic inhibition in probits and the logarithm of the concentration of the compound. Accordingly it is suggested that the best estimate of the activity of the compound is the concentration which produces 50 per cent mitotic inhibition under the experimental conditions. This concentration may be termed MI 50 as a convenient abbreviation. Among the related compounds studied (Mitchell and Simon-Reuss, 1952) most show a similar behaviour. An anomalous mitosis-concentration relation indicates mechanisms of biological action different from the quantal (all-or-nothing) response involved in the blockage of the entry of cells into mitosis. For example, a rapidly increasing prolongation of metaphase with concentration, at high concentrations of a compound may lead to a maximum of apparent mitotic inhibition ; this appears to occur in the case of Compound VIII (Mitchell and Simon-Reuss, 1952). It is of course necessary to study the mitotic phase distribution and cytological effects with any compound examined.

It is of great interest that with small doses of X-radiation under the present experimental conditions there is also no indication of departure from a linear relation between mitotic inhibition in probits and the logarithm of the dose of radiation.

The quantitative consideration of potentiation in the study of the effects of combinations of agents is a difficult problem of general biological importance. For the present purpose of selecting chemical agents as possible therapeutic radiosensitisers the summation method as applied here to the joint action of Compound I and small doses of X-radiation appears to be adequate and sufficiently simple for routine testing.

The first step is to select a compound which is a mitotic inhibitor, and with which the combined action of X-radiation shows potentiation of mitotic inhibition. However, mitotic inhibition is not the only factor involved, and probably it is not the most important factor to be considered. Studies of the mechanism of the therapeutic action of radiations and investigations of possible chemotherapeutic agents suggest that mitotic inhibition *per se* is not directly responsible for the retrogression of malignant tumours. There is much radiobiological evidence to support the view that the most important mechanism by which ionising radiations kill cells is chromosome breakage and reunion, with loss of chromosome parts and the production of inviable structural aberrations of the chromosomes. The relation between chromosome damage by radiation and the disturbance of the cellular metabolic processes is not understood. The role of chromosome structural changes in radiotherapy is, at present, uncertain, However, it seems plausible to expect potentiation of chromosome fragmentation in the combined action of X-radiation and of a chemical agent which may be a therapeutic radiosensitiser. This property is shown by Compound I. It seems reasonable to try to improve upon this compound by selecting compounds of related chemical structure, which alone produce more chromosome fragmentation, preferably together with anaphase bridges.

### SUMMARY.

1. More satisfactory quantitative experimental methods using the technique of tissue culture have been developed to study mitotic inhibition and radiosensitisation by chemical agents, using tetrasodium 2-methyl-1:4-naphthohydroquinone diphosphate (Compound I) as the reference substance.

2. The best estimate of the antimitotic activity of the compound is the concentration which produces 50 per cent mitotic inhibition under the experimental conditions. Studies of cytological effects are even more important than those of mitotic inhibition. Cell migration has been shown to be unimportant in these experiments.

3. For Compound I, there is no indication of departure from a linear relation between the mitotic inhibition in probits and the logarithm of the concentration.

For 50 per cent mitotic inhibition after 24 hours, the concentration is $3 \cdot 81 \pm 0 \cdot 15 \times 10^{-6}$M.

4. For X-radiation there is no indication of departure from a linear relation between mitotic inhibition in probits and the logarithm of the dose.

5. The study of the combination of the action of X-radiation and Compound I by means of the summation method has demonstrated potentiation of both mitotic inhibition and chromosome fragmentation.

6. Thermal instability of Compound I in aqueous solution in the absence of oxygen has been demonstrated. There is almost complete inactivation after incubation at 39° C. for 7 days. This finding may account for some erratic results in animal experiments and clinical trials.

We are indebted to Dr. F. Wrigley of Roche Products, Limited, Welwyn Garden City, for arranging supplies of " Synkavit".

### REFERENCES.

FINNEY, D. J.—(1947) 'Probit Analysis.' London (Cambridge University Press).

FISCHER, A., AND PARKER, R. C.—(1929) *Brit. J. exp. Path.*, **10**, 312.

FISHER, R. A., AND YATES, F.—(1948) 'Statistical Tables for Biological, Agricultural and Medical Research.' London (Oliver and Boyd).

GRAY, L. H., MOTTRAM, J. C., READ, J., AND SPEAR, F. G.—(1940) *Brit. J. Radiol.*, **13**, 371.

*Idem* AND READ, J.—(1944) *Ibid.*, **17**, 271.

GREBE, L., AND NITZGE, K.—(1930) 'Tabellen zur Dosierung der Roentgenstrahlen.' Berlin (Urban and Schwarzenberg).
GREENING, J. R.—(1947) *Brit. J. Radiol.*, **20**, 71.
HALDANE, J. B. S.—(1945) *Biometrika*, **33**, 222.
HUGHES, A. F. W.—(1949) *J. R. micr. Soc.*, **69**, 53.—(1952) *Exp. Cell Res.*, **3**, 108.
JACOBY, F.—(1937) *Arch. exp. Zellforsch.*, **19**, 241.
LAMERTON, L. F.—(1940) *Brit. J. Radiol.*, **13**, 136.
LASNITZKI, I., AND LEA, D. E.—(1940) *Ibid.*, **13**, 149.
LEVAN, A.—(1949) *Hereditas, Lund.* Suppl. Vol., 325.
LIECHTI, A., AND MULLER, J. H.—(1936) *Strahlentherapie*, **57**, 284.
MAYER, E.—(1939) *Tabul. Biol., Hague*, **19**, 65.
MITCHELL, J. S.—(1947) *Brit. J. Radiol.*, **20**, 368.—(1948) *Brit. J. Cancer*, **2**, 351.—(1949*a*) *Experientia*, **5**, 293.—(1949*b*) *Ann. Rep. Brit. Emp. Cancer Campgn.*, **27**, 214.—(1950) *Ibid.*, **28**, 213.—(1951) *Ibid.*, **29**, in press.
*Idem* AND SIMON-REUSS, I.—(1947) *Nature*, **160**, 98.—(1952) *Brit. J. Cancer*, **6**, 317.
PATERSON, E.—(1942) *Brit. J. Radiol.*, **15**, 257, 302.
SIMON-REUSS, I., AND SPEAR, F. G.—(1947) *Ibid.*, **20**, 63.
SPEAR, F. G.—(1935) *Ibid.*, **8**, 68, 280.
SPIERS, F. W.—(1949) *Ibid.*, **22**, 521.
WILLMER, E. N.—(1933) *J. exp. Biol.*, **10**, 323.